# Crystal structure and quantum-chemical calculations of a tri­methyl­aluminium–THF adduct

**DOI:** 10.1107/S2056989018001275

**Published:** 2018-01-31

**Authors:** Lukas Brieger, Andreas Hermann, Christian Unkelbach, Carsten Strohmann

**Affiliations:** a Technische Universität Dortmund, Anorganische Chemie, Otto-Hahn-Strasse 6, D-44227 Dortmund, Germany

**Keywords:** crystal structure, aluminium, tri­methyl­aluminium, thf, adduct, monomer, angle

## Abstract

The title compound, trimeth­yl(tetra­hydro­furan-κ*O*)aluminium(III), [Al(CH_3_)_3_(C_4_H_8_O)], is an addition product of tri­methyl­aluminium and tetra­hydro­furan (THF). Instead of a dimeric structure, which is very common for these types of compounds, a monomeric mol­ecular structure is observed. The C—Al—C angles in the mol­ecule are very different from the C—Al—C angles found in dimeric mol­ecular structures, leading to a different symmetry around the Al^III^ atom. The reasons for these differences are discussed.

## Abstract 2

The title compound, trimeth­yl(tetra­hydro­furan-κ*O*)aluminium(III), is an addition product of tri­methyl­aluminium and tetra­hydro­furan (THF). Instead of a dimeric structure, which is very common for these types of compounds, a monomeric mol­ecular structure is observed.

## Chemical context   

Similar to diborane, tri­methyl­aluminium is present as a dimer at room temperature. Analogously, two monomers are connected by two three-center–two-electron bonds bridging both mol­ecules. The monomer of tri­methyl­aluminium can be observed at high temperatures and low pressure with a trigonal-planar symmetry in the gas phase (Almenningen *et al.*, 1971 ▸). The most important use of tri­methyl­aluminium is in the production of methyl­aluminoxane, which is used to activate the Ziegler–Natta catalysts for olefin polymerization (Andresen *et al.*, 1976 ▸). Another known and important application is in the synthesis of Tebbe’s reagent, which is used for methyl­enation reactions in organic synthesis (Herrmann, 1982 ▸). The monomeric structure of tri­methyl­aluminium in THF could lead to a higher reactivity of this compound in these applications. Different THF adducts of tri­methyl­aluminium are known (Kong *et al.*, 1995 ▸; Vidyaratne *et al.*, 2009 ▸; Tanner *et al.*, 1993 ▸), but on the one hand the tri­methyl­aluminium–THF adduct is obtained as a co-crystallate and on the other it is part of a calcium sandwhich complex. Many ether adducts of AlMe_3_ are also known (see *e.g*. Robinson *et al.*, 1985 ▸, 1987 ▸; Zhao *et al.*, 1999 ▸; Leman *et al.*, 1993 ▸; Hsiao *et al.*, 2016 ▸; Zhang *et al.*, 1985 ▸; Atwood *et al.*, 1983 ▸). We decided to synthesize the THF adduct of tri­methyl­aluminium specifically to analyse its properties.
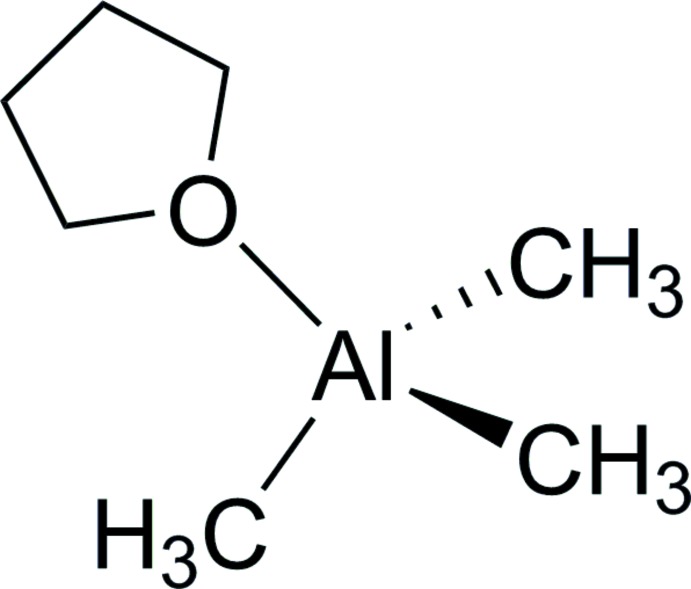


## Structural commentary   

**Figure 1:**
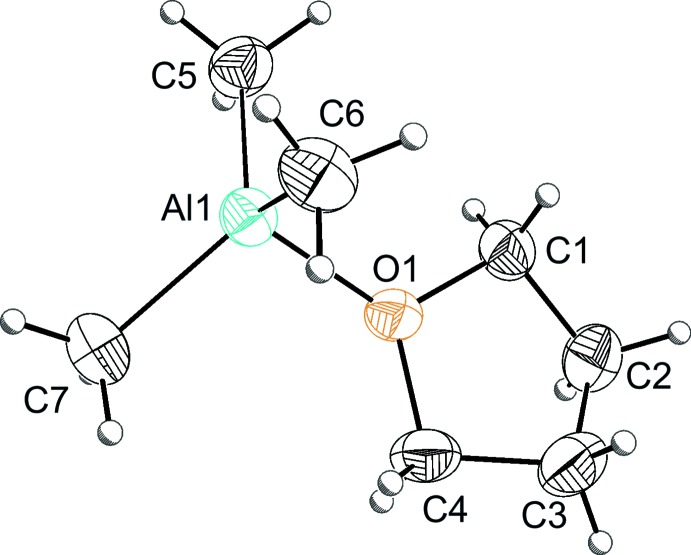
The mol­ecular structure of the title compound, with displacement ellipsoids drawn at the 50% probability level.

In the presence of THF, no dimeric tri­methyl­aluminium, but instead a monomeric tri­methyl­aluminium–thf adduct was formed. It was synthesized from a tri­methyl­aluminium solution in *n*-heptane under inert conditions. Crystals of this compound are pyrophoric, sensitive to moisture, oxygen and high temperature. As a consequence, they were handled and prepared at low temperature under an argon atmosphere. The title compound (Fig. 1 ▸) shows a structure between trigonal–planar and tetra­hedral symmetry, as indicated by the three C—Al—C angles [116.43 (9), 116.24 (9), 114.97 (9)°; Table 1 ▸]. As a result of the weak inter­action between aluminium and oxygen, an elongated Al—O bond distance is observed [1.9131 (13) Å]. The Al—O bond distance is very similar to those in known aluminium–THF adducts [Lehmkuhl *et al.*, 1985 ▸ (CSD recode DENGIM; Groom *et al.*, 2016 ▸); Schnitter *et al.*, 1997 ▸ (refcode NIKJEW)] and other mol­ecular structures where this compound could be obtained as a co-crystallate [Kong *et al.*, 1995 ▸ (YUGWEC); Vidyaratne *et al.*, 2009 ▸ (IHEYOK)]. Even longer Al—O bonds can be observed in the mol­ecular structure of a dioxane–tri­methyl­aluminium adduct [2.02 (2) Å; Atwood & Stucky, 1967 ▸ (TMALOX)] and in the mol­ecular structure of tri­methyl­aluminium with 18-crown-6 [2.005 (6) Å; Atwood *et al.*, 1982 ▸ (BOYVEQ)]. The dimeric aluminium structure (AFODUU) observed by Sharma *et al.* (2002 ▸) shows very short Al—O bonds for the terminal and bridging isoprop­oxy groups bound to the tetra-coordinated positions [1.799 (3)–1.685 (3) Å], indicating a strong inter­action between aluminium and oxygen in these cases. These results support the weak inter­action between aluminium and oxygen in the title compound, leading to a slight distortion of the tri­methyl­aluminium and a structure between trigonal–planar and tetra­hedral symmetry. In contrast to the monomeric structure, the dimeric mol­ecular structure of tri­methyl­aluminium features one large C—Al—C angle and two smaller C—Al—C angles [123.55 (1), 107.26 (1), 106.79 (1)°]. As a result of the lower electron density of the two bridging Al—C bonds, less space is required in the coordination sphere of the aluminium atom. In comparison with an ideal tetra­hedral coordination sphere, there is a smaller angle for the bridging bonds and a larger angle for the terminal bonds (Stammler *et al.*, 2015 ▸). These results are supported by further dimeric mol­ecular structures of tri­methyl­aluminium. Nikiforov *et al.* (2008 ▸) reported a titanium complex (BOFNOA) with a tri­methyl­aluminium unit with one large C—Al—C angle and two smaller C—Al—C angles [119.32 (13), 103.34 (13), 103.39 (12)° and 115.86 (13), 106.78 (13), 100.29 (12)°]. Occhipinti *et al.* (2011 ▸) reported the synthesis and stability of homoleptic metal(III) tetra­methyl­aluminates, which feature one large C—Al—C angle for the terminal bonds and two smaller C—Al—C angles for the bridging bonds. For the crystal strucutre of Tm(AlMe_4_)_3_, a large C—Al—C angle for the terminal bonds [118.55 (7) or 119.5 (2)°] and two smaller angles [104.84 (7), 108.64 (7)° or 105.43 (6), 108.86 (7)°] were observed.

## Quantum-chemical calculations   

Quantum-chemical calculations with the minnesota functional M062X, basis set 6–311+G(2df,p) and the solvent model (PCM) were applied to verify the C—Al and C—O bond lengths as well as the C—Al—C angles. Apparently, a simple gas-phase calculation is not sufficient to describe the crystal precisely, and leads to different results than found in the monomeric mol­ecular structure of this compound. Therefore we decided to use the solvent model (PCM). Götz *et al.* (2010 ▸) have recently evaluated the ability of an embedded-cluster model with polarizable continuum solvents (PCM) to reproduce the crystal environmental effects. Those models were used to reproduce the crystal environmental effects on QTAIM parameters inside the tetra­meric unit of solid MeLi (Götz *et al.*, 2013 ▸).

The NBO analysis of the Al—C bond indicates an occupancy of 82% for carbon and an occupancy of 18% for aluminium. The carbon part of the bond consists of 34% *s*-character and 66% *p*-character, whereas the aluminium part of the bond consists of 29% *s*-character, 70% *p*-character and 1% *d*-character. These results are in good agreement with the observed C—Al—C angles [116.43 (9), 116.24 (9), 114.97 (9)°]. The greater electronegativity of carbon provides more *p*-character in the Al—C bond, resulting in smaller angles than 120°. The NBO analysis for the Al—O bond indicates an occupancy of 94% for oxygen and an occupancy of 6% for aluminium. The oxygen part of the bond consists of 41% *s*-character and 59% *p*-character whereas the aluminium part of the bond consists of 14% *s*-character and 84% *p*-character and 2% *d*-character. The details of the NBO analysis are summarized in Table 2 ▸.

## Database survey   

The basic building unit, tri­methyl­aluminium, has been known for a long time and has been completely characterized (Lewis & Rundle, 1953 ▸; Vranka & Amma 1967 ▸). Studies using single crystal X-ray analysis have been made, determining the exact structure, and were found in a search of the Cambridge Structural Database (Groom *et al.*, 2016 ▸). A dimeric structure with bridging methyl groups can be obtained, which is formed by two monomers that share a center of symmetry. An inter­ior Al—C—Al angle of about 74° and an exterior C—Al—C angle of about 124° were observed. Furthermore the Al—C distances have significant differences. The Al—C bridging distance is 2.14 Å, whereas the Al—C terminal distance is about 2.00 Å. Tri­methyl­aluminium–THF adducts have been reported already. On the one hand this compound was observed as a co-crystallate of a self-activating ethyl­ene trimerization catalyst (IHEYOK; Vidyaratne *et al.*, 2009 ▸) and on the other hand as a co-crystallate of an unusual transition-metal cluster (YUGWEC; Kong *et al.*, 1995 ▸). The co-crystallate has not been further analysed.

## Synthesis and crystallization   

Tri­methyl­aluminium is predominately a dimer in hydro­carbon solution. The structure was obtained treating 200 mg (1.38 mmol, 1.0 eq.) of a 2 *M* solution of tri­methyl­aluminium in *n*-heptane with 0.08 ml (1.38 mmol, 1.0 eq.) THF under inert conditions. The sample was stored under an argon atmosphere at 193 K for two weeks and crystallized as colourless blocks. The yield was not determined. The crystals are pyrophoric and were prepared with the help of ‘X-Temp 2′ (Heine & Stalke, 1992 ▸; Stalke, 1998 ▸). Further methods of analysis or characterization were not possible because of the high reactivity of this compound.

## Refinement   

Crystal data, data collection and structure refinement details are summarized in Table 3 ▸. The C-bound H atoms were included in calculated positions and treated as riding atoms, with C—H = 0.98 Å, *U*_iso_(H) = 1.5*U*_eq_(C) for methyl hydrogen atoms and C—H = 0.99 Å, *U*_iso_(H) = 1.2*U*_eq_(C) for methyl­ene hydrogen atoms.

## Supplementary Material

Crystal structure: contains datablock(s) I. DOI: 10.1107/S2056989018001275/lh5865sup1.cif

Structure factors: contains datablock(s) I. DOI: 10.1107/S2056989018001275/lh5865Isup2.hkl

CCDC reference: 1588683

Additional supporting information:  crystallographic information; 3D view; checkCIF report

## Figures and Tables

**Table 1:** Selected geometric parameters (Å, °)

O1—Al1	1.9131 (13)	C6—Al1	1.976 (2)
C5—Al1	1.9671 (19)	C7—Al1	1.965 (2)
			
C5—Al1—C6	116.43 (9)	C1—O1—Al1	122.45 (10)
C5—Al1—C7	116.24 (9)	C4—O1—Al1	124.00 (11)
C6—Al1—C7	114.97 (9)	C4—O1—C1	109.40 (13)

**Table 2:** NBO analysis

Al—O	Al	O	Al—C	Al	C
Occupancy	5.70%	94.30%		18.22%	81.78%
*s*	14.29%	41.14%		28.82%	33.59%
*p*	83.68%	58.84%		70.24%	66.39%
*d*	1.95%	0.01%		0.91%	0.01%

**Table 3:** Experimental details

Crystal data	
Chemical formula	[Al(CH_3_)_3_(C_4_H_8_O)]
*M*_r_	144.18
Crystal system, space group	Monoclinic, *P*2_1_/*c*
Temperature (K)	150
*a*, *b*, *c* (Å)	7.7034 (6), 9.1228 (7), 13.6833 (11)
β (°)	98.845 (8)
*V* (Å^3^)	950.18 (13)
*Z*	4
Radiation type	Mo *K*α
μ (mm^−1^)	0.15
Crystal size (mm)	0.4 × 0.4 × 0.2

Data collection	
Diffractometer	Oxford Diffraction Xcalibur Sapphire3
Absorption correction	Multi-scan (*CrysAlis PRO*; Oxford Diffraction, 2010 ▸)
*T*_min_, *T*_max_	0.767, 0.971
No. of measured, independent and observed [*I* > 2σ(*I*)] reflections	16552, 2071, 1664
*R*_int_	0.085
(sin θ/λ)_max_ (Å^−1^)	0.639

Refinement	
*R*[*F* ^2^ > 2σ(*F* ^2^)], *wR*(*F* ^2^), *S*	0.054, 0.152, 1.09
No. of reflections	2071
No. of parameters	85
H-atom treatment	H-atom parameters constrained
Δρ_max_, Δρ_min_ (e Å^−3^)	0.89, −0.33

Computer programs: *CrysAlis PRO* (Oxford Diffraction, 2010 ▸), *SIR2004* (Burla *et al.*, 2007 ▸), *SHELXL2014* (Sheldrick, 2015 ▸) and *OLEX2* (Dolomanov *et al.*, 2009 ▸).

## supplementary crystallographic information

### Crystal data

**Table:**

[Al(CH_3_)_3_(C_4_H_8_O)]	*F*(000) = 320
*M**_r_* = 144.18	*D*_x_ = 1.008 Mg m^−^^3^
Monoclinic, *P*2_1_/*c*	Mo *K*α radiation, λ = 0.71073 Å
*a* = 7.7034 (6) Å	Cell parameters from 8661 reflections
*b* = 9.1228 (7) Å	θ = 2.2–29.4°
*c* = 13.6833 (11) Å	µ = 0.15 mm^−^^1^
β = 98.845 (8)°	*T* = 150 K
*V* = 950.18 (13) Å^3^	Block, colourless
*Z* = 4	0.4 × 0.4 × 0.2 mm

### Data collection

**Table:**

Oxford Diffraction Xcalibur Sapphire3 diffractometer	2071 independent reflections
Radiation source: Enhance (Mo) X-ray Source	1664 reflections with *I* > 2σ(*I*)
Graphite monochromator	*R*_int_ = 0.085
Detector resolution: 16.0560 pixels mm^-1^	θ_max_ = 27.0°, θ_min_ = 2.7°
ω scans	*h* = −9→9
Absorption correction: multi-scan (CrysAlis PRO; Oxford Diffraction, 2010)	*k* = −11→11
*T*_min_ = 0.767, *T*_max_ = 0.971	*l* = −17→17
16552 measured reflections	

### Refinement

**Table:**

Refinement on *F*^2^	Primary atom site location: structure-invariant direct methods
Least-squares matrix: full	Hydrogen site location: inferred from neighbouring sites
*R*[*F*^2^ > 2σ(*F*^2^)] = 0.054	H-atom parameters constrained
*wR*(*F*^2^) = 0.152	*w* = 1/[σ^2^(*F*_o_^2^) + (0.101*P*)^2^ + 0.0468*P*] where *P* = (*F*_o_^2^ + 2*F*_c_^2^)/3
*S* = 1.09	(Δ/σ)_max_ = 0.001
2071 reflections	Δρ_max_ = 0.89 e Å^−^^3^
85 parameters	Δρ_min_ = −0.32 e Å^−^^3^
0 restraints	

### Special details

**Table:**

Geometry. All esds (except the esd in the dihedral angle between two l.s. planes) are estimated using the full covariance matrix. The cell esds are taken into account individually in the estimation of esds in distances, angles and torsion angles; correlations between esds in cell parameters are only used when they are defined by crystal symmetry. An approximate (isotropic) treatment of cell esds is used for estimating esds involving l.s. planes.

### Fractional atomic coordinates and isotropic or equivalent isotropic displacement parameters (Å^2^)

**Table:**

	*x*	*y*	*z*	*U*_iso_*/*U*_eq_	
O1	0.23608 (15)	0.37473 (13)	0.62125 (9)	0.0358 (3)	
C1	0.4234 (2)	0.3761 (2)	0.61379 (15)	0.0403 (4)	
H1A	0.4953	0.3839	0.6801	0.048*	
H1B	0.4521	0.4592	0.5726	0.048*	
C2	0.4549 (3)	0.2322 (2)	0.56601 (16)	0.0500 (5)	
H2A	0.5778	0.1990	0.5853	0.060*	
H2B	0.4284	0.2389	0.4930	0.060*	
C3	0.3280 (3)	0.1316 (2)	0.60652 (16)	0.0507 (5)	
H3A	0.3019	0.0440	0.5642	0.061*	
H3B	0.3748	0.1000	0.6747	0.061*	
C4	0.1680 (3)	0.2259 (2)	0.60476 (16)	0.0445 (5)	
H4B	0.0906	0.2184	0.5401	0.053*	
H4A	0.1006	0.1966	0.6576	0.053*	
C5	0.2460 (3)	0.7005 (2)	0.65333 (15)	0.0439 (5)	
H5A	0.3731	0.6915	0.6742	0.066*	
H5B	0.2022	0.7858	0.6856	0.066*	
H5C	0.2212	0.7130	0.5814	0.066*	
C6	0.1905 (3)	0.4582 (2)	0.83000 (15)	0.0461 (5)	
H6A	0.1225	0.3705	0.8410	0.069*	
H6B	0.1635	0.5367	0.8742	0.069*	
H6C	0.3162	0.4354	0.8438	0.069*	
C7	−0.1208 (3)	0.5008 (2)	0.63514 (15)	0.0436 (5)	
H7A	−0.1357	0.5156	0.5634	0.065*	
H7B	−0.1902	0.5737	0.6648	0.065*	
H7C	−0.1608	0.4021	0.6493	0.065*	
Al1	0.12856 (7)	0.52245 (6)	0.69114 (4)	0.0335 (2)	

### Atomic displacement parameters (Å^2^)

**Table:**

	*U*^11^	*U*^22^	*U*^33^	*U*^12^	*U*^13^	*U*^23^
O1	0.0322 (7)	0.0311 (6)	0.0447 (7)	−0.0050 (5)	0.0082 (5)	−0.0018 (5)
C1	0.0311 (9)	0.0408 (10)	0.0500 (11)	−0.0005 (7)	0.0088 (7)	0.0007 (8)
C2	0.0481 (11)	0.0471 (12)	0.0560 (13)	0.0081 (9)	0.0119 (9)	−0.0046 (9)
C3	0.0624 (14)	0.0336 (10)	0.0554 (13)	0.0024 (9)	0.0067 (10)	0.0011 (8)
C4	0.0451 (10)	0.0351 (10)	0.0535 (12)	−0.0111 (8)	0.0087 (8)	−0.0039 (8)
C5	0.0456 (11)	0.0368 (10)	0.0508 (12)	−0.0022 (8)	0.0117 (8)	0.0014 (8)
C6	0.0474 (11)	0.0526 (12)	0.0368 (10)	−0.0045 (9)	0.0021 (8)	0.0038 (8)
C7	0.0342 (10)	0.0541 (12)	0.0419 (11)	0.0013 (8)	0.0037 (8)	0.0035 (8)
Al1	0.0318 (3)	0.0352 (3)	0.0336 (3)	−0.0007 (2)	0.0048 (2)	0.00140 (19)

### Geometric parameters (Å, º)

**Table:**

O1—C1	1.462 (2)	C3—C4	1.500 (3)
O1—C4	1.460 (2)	C5—Al1	1.9671 (19)
O1—Al1	1.9131 (13)	C6—Al1	1.976 (2)
C1—C2	1.503 (3)	C7—Al1	1.965 (2)
C2—C3	1.508 (3)		
			
O1—C1—C2	104.49 (15)	C5—Al1—C6	116.43 (9)
C1—C2—C3	102.38 (16)	C5—Al1—C7	116.24 (9)
C4—C3—C2	102.87 (16)	C6—Al1—C7	114.97 (9)
O1—C4—C3	104.82 (15)	C1—O1—Al1	122.45 (10)
O1—Al1—C5	101.44 (7)	C4—O1—Al1	124.00 (11)
O1—Al1—C6	102.27 (8)	C4—O1—C1	109.40 (13)
O1—Al1—C7	101.92 (8)		

### Selected geometric parameters (Å, °).

**Table:**

O1–Al1		1.9131 (13)
C5–Al1		1.9671 (19)
C6–Al1		1.976 (2)
C7–Al1		1.965 (2)
		
C5–Al1–C6		116.43 (9)
C7–Al1–C5		116.24 (9)
C7–Al1–C6		114.97 (9)
C1–O1–Al1		122.45 (10)
C4–O1–Al1		124.00 (11)
C4–O1–C1		109.40 (13)
